# MGeND: an integrated database for Japanese clinical and genomic information

**DOI:** 10.1038/s41439-019-0084-4

**Published:** 2019-12-06

**Authors:** Mayumi Kamada, Masahiko Nakatsui, Ryosuke Kojima, Sachio Nohara, Eiichiro Uchino, Shigeki Tanishima, Masaya Sugiyama, Kenjiro Kosaki, Katsushi Tokunaga, Masashi Mizokami, Yasushi Okuno

**Affiliations:** 1 Department of Biomedical Data Intelligence, Graduate School of Medicine, Kyoto University, 53 Shogoin-Kawaharacho. Sakyo-ku, Kyoto, 606-8507 Japan; 2 Mitsubishi Space Software Co., Ltd., 5-4-36 Tsukaguchi-honmachi, Amagasaki, Hyogo 661-0001 Japan; 3 The Research Center for Hepatitis and Immunology, National Center for Global Health and Medicine, 1-7-1 Kohnodai, Ichikawa, Chiba 272-8516 Japan; 4 Center for Medical Genetics, Keio University School of Medicine, 35 Shinanomachi, Shinjuku-ku, Tokyo 160-8582 Japan; 5 Department of Human Genetics, Graduate School of Medicine, The University of Tokyo, 7-3-1 Hongo, Bunkyo-ku, Tokyo 113-0033 Japan; 6 Present Address: Genome Medical Science Project (Toyama), National Center for Global Health and Medicine, 1-21-1 Toyama, Shinjuku-ku, Tokyo 162-8655 Japan

**Keywords:** Genetic databases, Genetic variation

## Abstract

To promote the implementation of genomic medicine, we developed an integrated database, the Medical Genomics Japan Variant Database (MGeND). In its first release, MGeND provides data regarding genomic variations in Japanese individuals, collected by research groups in five disease fields. These variations consist of curated SNV/INDEL variants and susceptibility variants for diseases established by genome-wide association study analysis. Furthermore, we recorded the frequencies of HLA alleles in infectious disease populations.

The accumulation of data regarding associations between genotypes and clinical phenotypes is important to accelerate the implementation of genomic medicine in clinical practice. Several databases containing genetic information and their clinical significance have already been released. ClinVar, developed by the National Institutes of Health in the US, provides genomic variant information with supporting evidence and review status^1^ and is widely utilized for the clinical interpretation of variants. Furthermore, some databases provide variant information regarding specific diseases.

There are two major problems with the utilization of databases for genomic medicine. The first pertains to the differences between populations. The genomic information stored in previously established databases has been primarily obtained from US and European populations. Genes and genotypes associated with the risk of onset of several diseases have been reported to vary between ethnic groups^2^. The second is the disease fields of the databases. Certain diseases are known to be triggers for other diseases, such as hepatitis and cancer^3^, and an example in which rare variants contribute to the risk of common diseases has been reported^4^. Interpretation of variants across diseases is necessary to elucidate variants and diseases with unknown mechanisms. However, there is no database of clinical and genomic information that reflects the characteristics of Asian populations across multiple disease fields, including monogenic and polygenic diseases.

We developed a database, the Medical Genomics Japan Variant Database, “MGeND”, which integrates clinical and genomic information regarding Japanese individuals. The first version of MGeND was released in March 2018, with genomic variations collected from 11 representative Japanese groups in the fields of “cancer”, “rare/intractable disease”, “dementia”, “infectious disease”, and “hearing loss”. The research groups in each disease field recruited patients and performed genomic analysis and interpretation of variants (Supplementary Table 1 presents the list of research groups). In collaboration with these groups, we collected and integrated genomic and clinical information that can be publicly shared on MGeND.

The clinical data to be registered include disease or diagnosis name along with basic patient background information, such as sex and age, excluding information that could identify individuals. Disease names are registered using general condition identifiers, such as Online Mendelian Inheritance in Man^5^, Human Phenotype Ontology^6^, and ICD10 (ref. ^7^). The age of onset and age at which the test was conducted can be submitted based on the disease type, with age divided into 10-year age bins in MGeND.

Because different genomic analyses can be conducted in monogenic and polygenic diseases, varying genomic data can be submitted to MGeND. Therefore, submission data formats have been defined for each genomic data type. In the first release of MGeND, we provided SNV/INDEL variants, susceptibility variants obtained by genome-wide association study (GWAS) analysis, and human leukocyte antigen (HLA) allele frequencies. To submit sequence variants, a valid description of a variant consists of a set of chromosome coordinates, changes, and the assembly version. Each variant position submitted is integrated into the GRCh38/hg38 assembly to be combined with public databases.

Furthermore, we accept sets of susceptibility variants identified using GWAS analysis often performed for some diseases, particularly for polygenic diseases. The statistical criteria of the data to be submitted are based on the judgment of the submitters. We recommend submitting variants with a *p*-value < 10^−4^.

Protein molecules encoded by HLA genes play key roles in the immune system, including antigen presentation and self-recognition. Accordingly, it is important to know the HLA types not only for autoimmune and infectious diseases but also for cancer. Therefore, we accept HLA allele frequency data represented in two or three/four fields. Currently, these types of variant data are not included in ClinVar and other databases.

In addition, for all types of variations, we recommend submitting information regarding details such as platform type, gene panels, methods, statistical tests, and imputation methods used for genotyping. In particular, for SNV/INDEL variants, we suggest that research groups submit variants with evidence for clinical significance and curation; MGeND provides publication information (PubMed ID) for each submission if it is available. Table 1 shows the number of variants for each data type in each disease field published in MGeND as of 16 February 2019.

**Table 1:** Number of variants registered in MGeND as of 16 February 2019.

Data type	Variants^*^	GWAS	HLA allele data
Disease field			
Cancer	16,685 (5550)	–	–
Rare/intractable disease	2711 (1920)	–	–
Infectious disease	19 (19)	155,098 (754)	1821 (841)
Hearing loss	122 (122)	–	–
Dementia	7682 (1669) APOE gene: 12,298 (5196)	410 (410)	–

The numbers within parentheses indicate the number of published variants. Variants that have not been released will be published when the date set by the submitter is approached. Based on the Japan Agency for Medical Research and Development (AMED) data sharing policy, submitters can leave their data unpublished until 2 years after analysis is complete or until a journal regarding the variant is published

To interpret variants, it is necessary to make comprehensive judgments by searching related information from a huge amount of data stored in public databases. Thus, the web display of MGeND has been designed to support the clinical interpretation of variants for medical research and clinical use.

Users can search variant information in MGeND using free text, such as disease name, gene symbol, or genomic position. The list of variants produced by the keyword search is displayed, with the clinical significance identified by submitters and information regarding public databases that are often used for clinical interpretation (Fig. 1a). Furthermore, investigation of diseases, drugs, and genes associated with the query is possible using the filters in the side bar. The list of all public databases displayed in MGeND is shown in Supplementary Table 2.

**Fig. 1:**
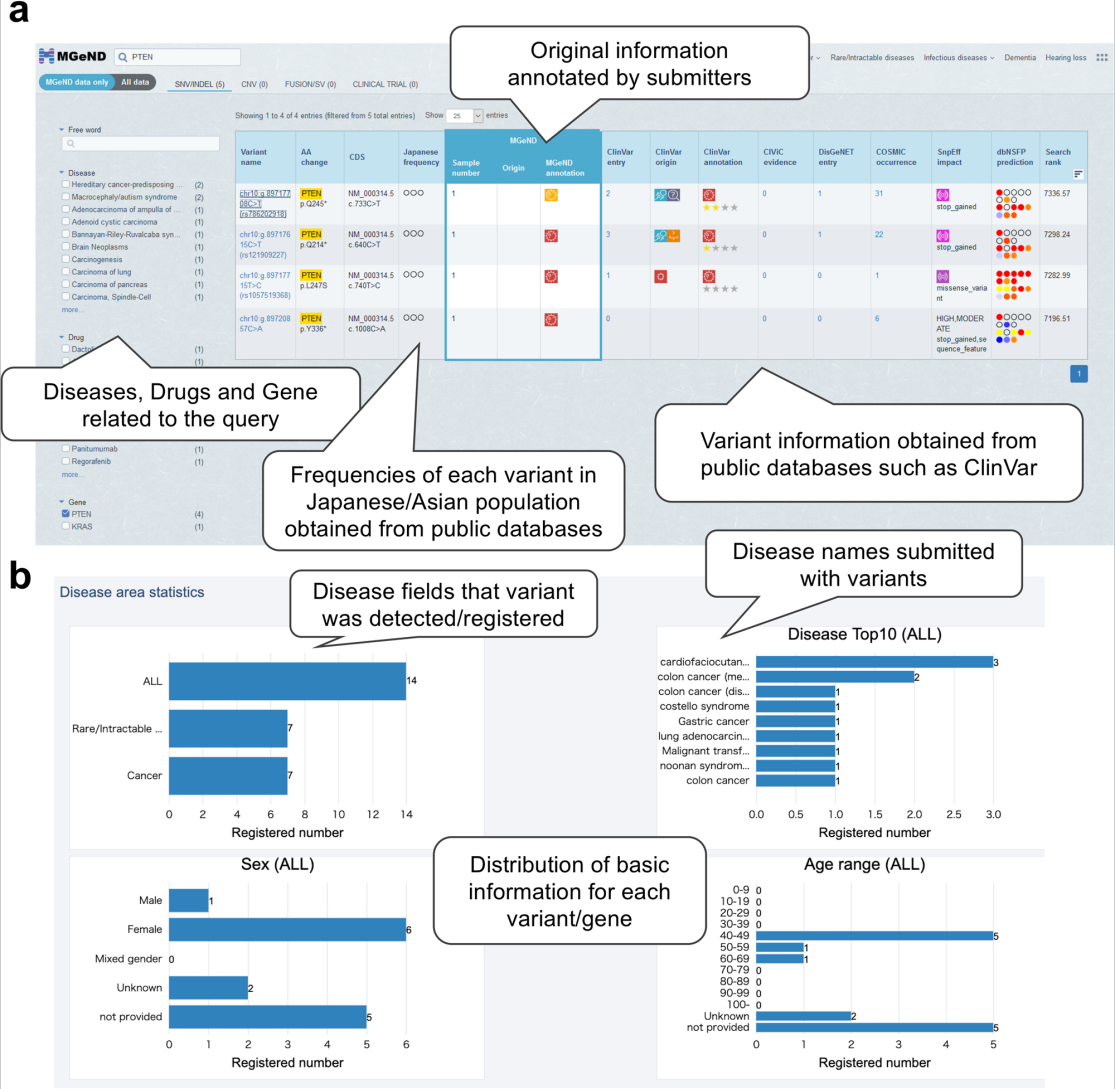
Graphical views of MGeND. **a** The resulting view from a keyword search. The variants identified by the keyword search are listed with the original information in MGeND (clinical significance, the number of samples, and origin of cells) and with the information from public databases, which is often used for interpretation of variants. **b** The statistics for each gene or variant are provided. The statistics regarding disease name, sex, and age can be filtered in each disease field by clicking on the disease field name in the upper left graph. Full text of the disease name is shown by placing the cursor over the disease name at the left side of the bar.

After selecting a variant or gene in the list of search results, detailed information can be obtained from the variant or gene pages. Each variant or gene page provides information about the disease fields and disease name for which that variant was reported and age and sex distributions of cases in which the variant was detected (Fig. 1b).

There are some variants common to different diseases, and analyzing these variants can assist in clarifying the underlying disease mechanisms. For example, a variant in the MAP2K1 gene (NC_000015.9:g.66727483G>A) is known to be associated with cardiofaciocutaneous syndrome 1 and cancer. In MGeND, the variant has been submitted by groups researching cancer and rare diseases, and users can confirm the situation on the variant page (details are provided in the Supplementary Material).

Furthermore, we provide specific viewers for some disease fields. For infectious diseases, we implemented a table viewer for the frequencies of HLA alleles in each study, with the frequencies of each allele in the healthy control group obtained from studies performed by the National Center for Global Health and Medicine and the HLA Laboratory^8^ (Fig. 2a). The APOE gene is known to be associated with the risk of onset of dementia^9^. Thus, the data submitted by the groups researching dementia can be filtered by type of dementia, sex, family history, and diagnosis source; the frequencies of the genotypes in the selected data are shown as pie graphs on the dementia page (Fig. 2b).

**Fig. 2:**
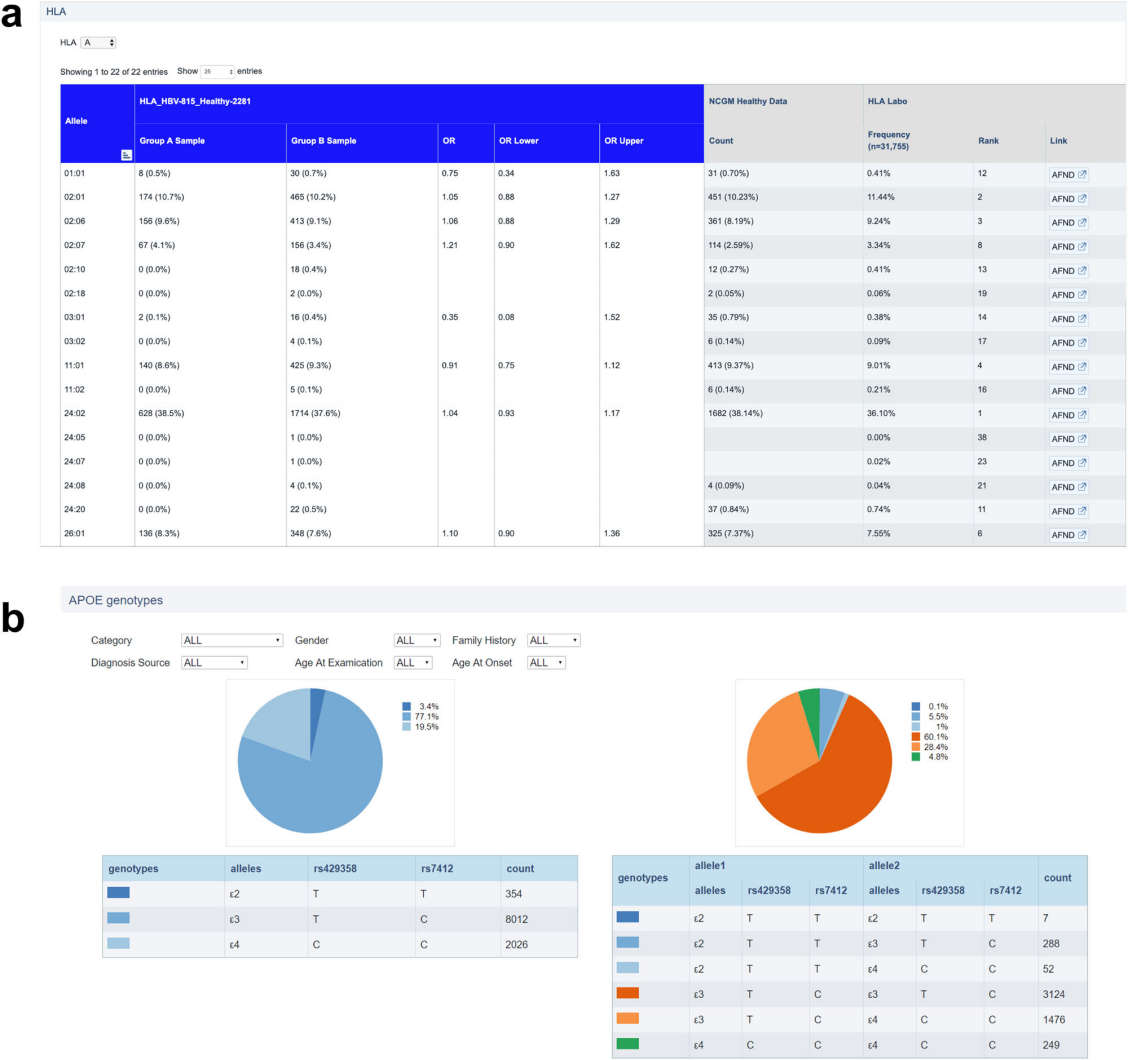
Specific viewers for infectious diseases and dementia. **a** The table viewer for the frequencies of human leukocyte antigen (HLA) alleles. The columns with blue headers are the HLA allele frequencies in patients and control samples for each study of the infectious disease group. The gray columns display the frequencies in the healthy control groups obtained from HLA Laboratory and National Center for Global Health and Medicine (NCGM) studies. **b** The graph for the genotype distribution of the APOE gene. The data used to draw these graphs can be filtered by factors such as sex, family history, and age of onset.

MGeND is the first database that provides disease-related genomic information specific to Asian populations and integrates variant information from monogenic and polygenic diseases. We aim to develop pages and contents that can present variant information more usefully; for example, we are developing the viewer for the GWAS dataset with meta-data, such as study design and *p*-values. We aim to expand and integrate the disease fields and accept submissions from researchers in various fields. The number of variants recorded in the database is expected to increase continuously.

## Supplementary information

Supplemental material

## Data Availability

MGeND is available from the following URL: https://mgend.med.kyoto-u.ac.jp.
