# Supplementary material for: MGeND: an integrated database for Japanese clinical and genomic information
Source: Hum Genome Var. 2019 Dec 6;6:53. doi: 10.1038/s41439-019-0084-4 (PMC6897987; doi:10.1038/s41439-019-0084-4)
Supplement: Supplementary file 1 — Supplemental material [file 41439_2019_84_MOESM1_ESM.docx]

*Human Genome Variation*

**Supplementary Information**

**Submittable data to MGeND**

The submittable genomic data is the followings; i) the data obtained Informed Consent (IC) from patient including the contents that the analysis results will be submitted to public database or published in research paper, ii) the data already published in academically trusted media, such as research paper and public database. Whether data registration is possible or not is confirmed by MGeND Registration Management Committee based on the research proposal, the notification from research ethics committee and the patient consent form submitted from each institute.

**Variants shared with multiple diseases**

The variant page in MGeND provides information about the disease name and field that each variant was detected. By looking at “Disease area statistics” on each variant page, you can find variant shared with multiple disease fields.

The following shows how to search for variant introduced in the main text. User can search other variants that shared with multiple diseases using the same steps.

1. Enter a text *“NC_000015.9:g.66727483G>A”* in search box on the top page, and click “Search” icon next to the box.

**
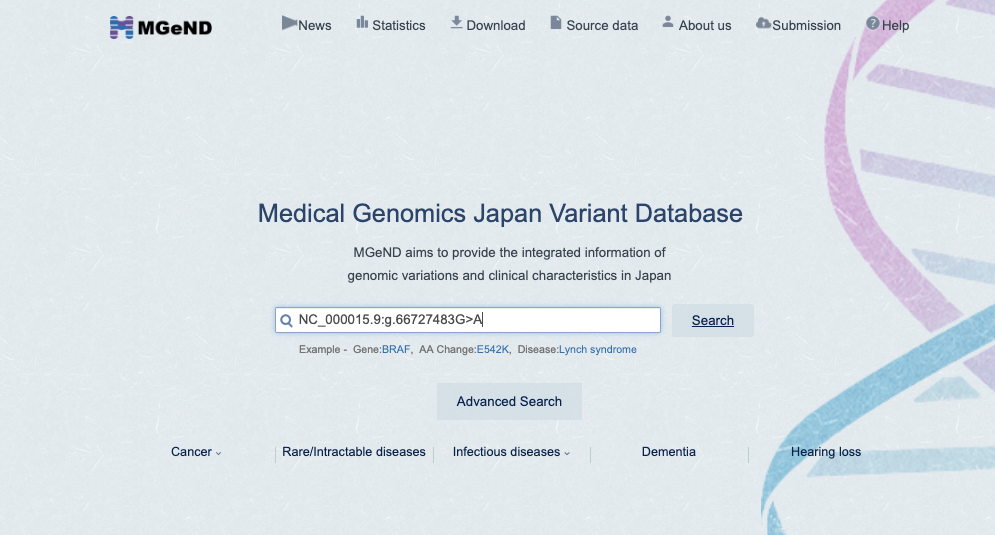
**

2. Click a link of variant name “NC_000015.9:g.66727483G>A (rs727504317)” in the result table.

**
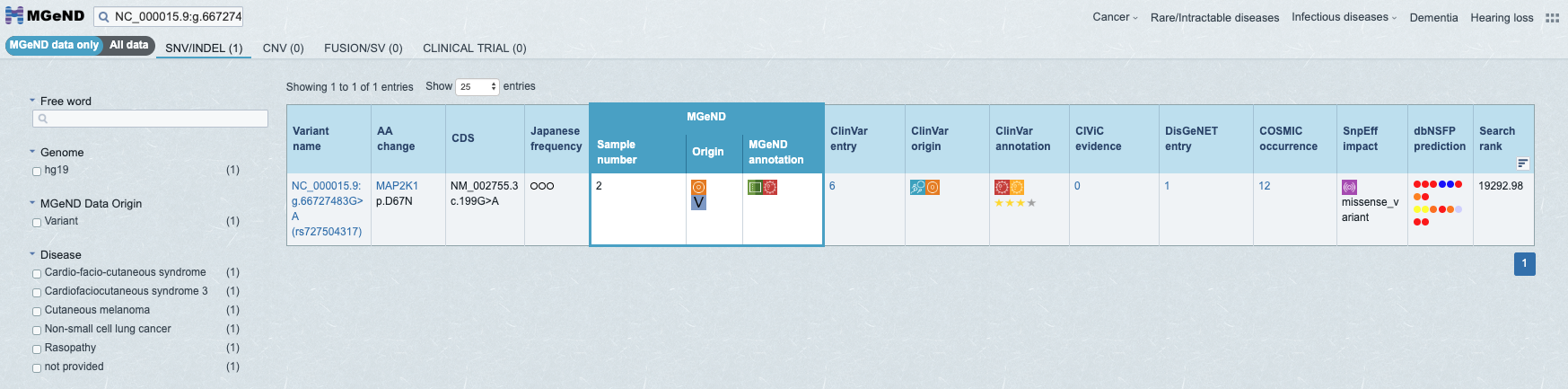
**

3. Check a field of “Disease are statistics” on the variant detail page. User can confirm that this variant has been registered from cancer field and rare/intractable disease field in the bar graph. Full text of disease name is shown by placing the cursor over disease name at the left side of bar.

**
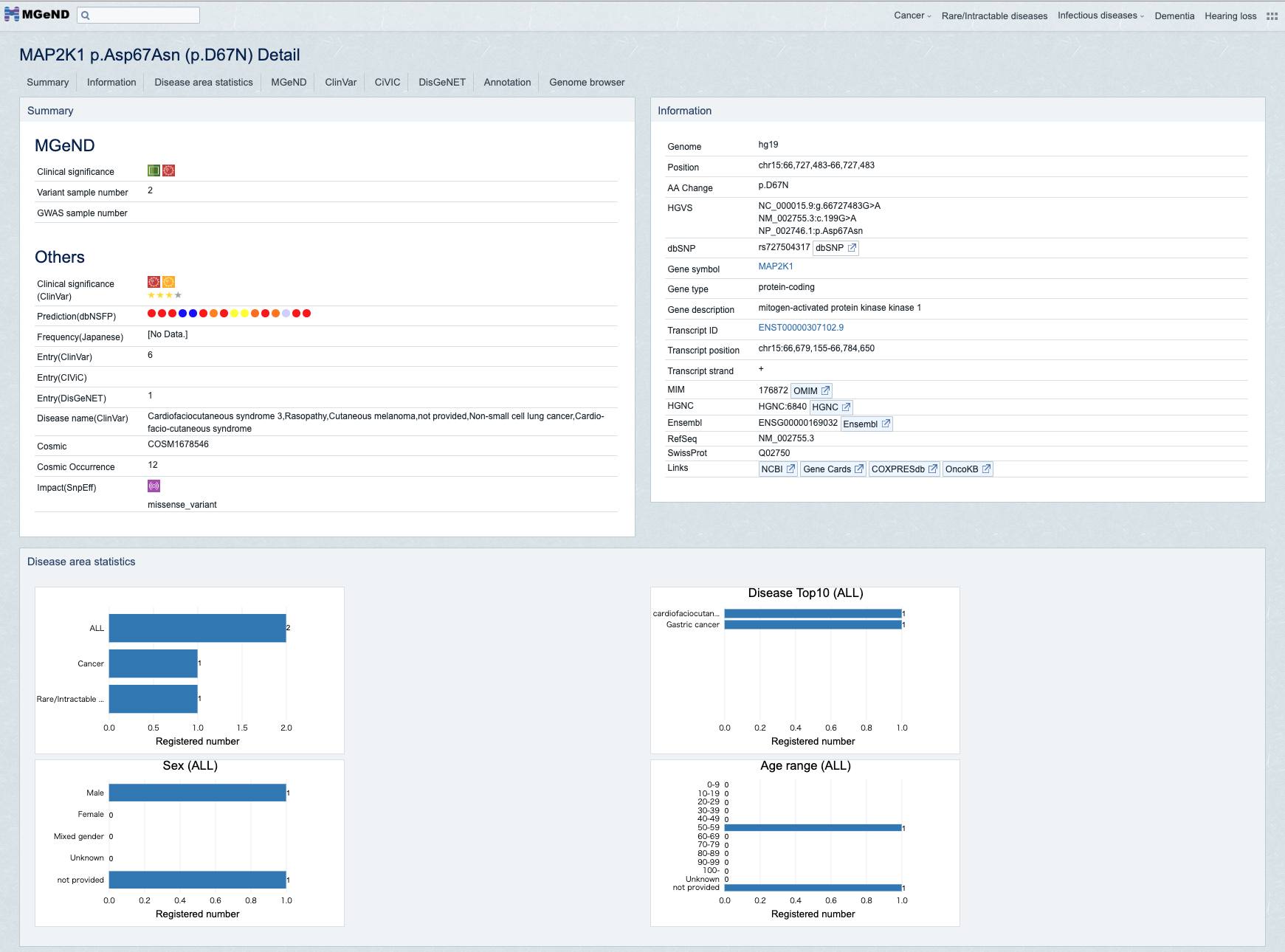
**

**Supplementary Table 1**

The list of research groups in the "Program for an Integrated Database of Clinical and Genomic Information" has been launched by AMED.

| Disease area | Representative Institute |
| --- | --- |
| Rare/Intractable disease | Keio University Group |
|  | The University of Tokyo Group |
| Cancer | National Cancer Center Hospital Group |
|  | Nagoya Medical Center Group |
|  | The University of Tokyo Group |
|  | Kyoto University Group |
| Infectious disease | Kyoto University Group |
|  | National Center for Global Health and Medicine Group |
|  | National Institute of Infectious Disease Group |
| Dementia | Osaka City University Group |
| Hearing loss | Shinshu University Group |

**Supplementary Table 2**

The list of databases integrated and displayed in MGeND (as of January 21, 2019)

| Name | Version | Url |
| --- | --- | --- |
| NCBI Gene Info | 20180727 | <ftp://ftp.ncbi.nlm.nih.gov/gene/DATA/gene_info.gz> |
| GenCode Transcript  (hg19 overlifted) | 28 | [https://www.gencodegenes.org](https://www.gencodegenes.org/) |
| GenCode Transcript | 28 | [https://www.gencodegenes.org](https://www.gencodegenes.org/) |
| Gencode transcript metadata | 28 | <https://www.gencodegenes.org/releases/current.html> |
| Disease Ontology | 20180620 | <https://sourceforge.net/p/diseaseontology/code/HEAD/tree/trunk/HumanDO.obo?format=raw> |
| dbSNP VCF | b150 |  |
| dbSNP 150 | b150 | <ftp://ftp.ncbi.nih.gov/snp/organisms/human_9606_b151_GRCh37p13/VCF/All_20180423.vcf.gz> |
| HGVD | 2.30 | <http://www.hgvd.genome.med.kyoto-u.ac.jp/HGVD1210-V2_30-dbSNP150.tar.gz> |
| ToMMo SNP | 1 | <http://humandbs.biosciencedbc.jp/files/hum0015/hum0015.v1.freq.v1.zip> |
| ExAC | 1 | <ftp://ftp.broadinstitute.org/pub/ExAC_release/release1/ExAC.r1.sites.vep.vcf.gz> |
| MMMP | 20160818 | <http://www.mmmp.org/mmmpFile/TargetedTherapyDatabase_TTD3_published.xls> |
| DisGeNET | 4.0 | <http://www.disgenet.org/web/DisGeNET/menu> |
| Drug list of Screening Committee of Anticancer Drugs | 20160209 | <https://scads.jfcr.or.jp/db/table.html> |
| CIViC Variant Summaries | 20180727-nightly |  |
| CIViC Evidence | 20180727-nightly |  |
| GWAS EFO Mapping | 20180829 | <ftp://ftp.ebi.ac.uk/pub/databases/gwas/releases/2018/08/29/gwas-efo-trait-mappings.tsv> |
| GWAS Catalog | 20180829 | <ftp://ftp.ebi.ac.uk/pub/databases/gwas/releases/2018/08/29/gwas-catalog-associations.tsv> |
| SnpEff result (hg19 variants) |  |  |
| SnpEff result (hg38 variants) |  |  |
| OMIM | 20180523 |  |
| Human Phenotype Ontology | 2018-07-25 | [https://hpo.jax.org](https://hpo.jax.org/) |
| OrphaNet | V2.6 | <https://www.orpha.net/> |
| MedGen Concept name | 20180516 |  |
| Insert disease from ICD10 |  |  |
| MeSH  (Medical Subject Headings) | 20180711 | <https://www.ncbi.nlm.nih.gov/mesh> |
| ClinVar | 2018-07 | <https://www.ncbi.nlm.nih.gov/clinvar/> |
| cosmic_occurrence | 85 | <https://cancer.sanger.ac.uk/cosmic> |
